# The Combined Effect of Individual and Neighborhood Socioeconomic Status on Cancer Survival Rates

**DOI:** 10.1371/journal.pone.0044325

**Published:** 2012-08-30

**Authors:** Chun-Ming Chang, Yu-Chieh Su, Ning-Sheng Lai, Kuang-Yung Huang, Sou-Hsin Chien, Yu-Han Chang, Wei-Cheng Lian, Ta-Wen Hsu, Ching-Chih Lee

**Affiliations:** 1 Department of Surgery, Buddhist Dalin Tzu Chi General Hospital, Chiayi, Taiwan; 2 School of Medicine, Tzu Chi University, Hualian, Taiwan; 3 Division of Hematology-Oncology, Department of Internal Medicine, Buddhist Dalin Tzu Chi General Hospital, Chiayi, Taiwan; 4 Cancer Center, Buddhist Dalin Tzu Chi General Hospital, Chiayi, Taiwan; 5 Division of Rheumatology, Department of Internal Medicine, Buddhist Dalin Tzu Chi General Hospital, Chiayi, Taiwan; 6 Department of Medical Research, Buddhist Dalin Tzu Chi General Hospital, Chiayi, Taiwan; 7 Division of Metabolism and Endocrinology, Department of Internal Medicine, Buddhist Dalin Tzu Chi General Hospital, Chiayi, Taiwan; 8 Department of Otolaryngology, Buddhist Dalin Tzu Chi General Hospital, Chiayi, Taiwan; 9 Center for Clinical Epidemiology and Biostatistics, Buddhist Dalin Tzu Chi General Hospital, Chiayi, Taiwan; 10 Community Medicine Research Center and the Institute of Public Health, National Yang-Ming University, Taipei, Taiwan; University of Nebraska Medical Center, United States of America

## Abstract

**Background:**

This population-based study investigated the relationship between individual and neighborhood socioeconomic status (SES) and mortality rates for major cancers in Taiwan.

**Methods:**

A population-based follow-up study was conducted with 20,488 cancer patients diagnosed in 2002. Each patient was traced to death or for 5 years. The individual income-related insurance payment amount was used as a proxy measure of individual SES for patients. Neighborhood SES was defined by income, and neighborhoods were grouped as living in advantaged or disadvantaged areas. The Cox proportional hazards model was used to compare the death-free survival rates between the different SES groups after adjusting for possible confounding and risk factors.

**Results:**

After adjusting for patient characteristics (age, gender, Charlson Comorbidity Index Score, urbanization, and area of residence), tumor extent, treatment modalities (operation and adjuvant therapy), and hospital characteristics (ownership and teaching level), colorectal cancer, and head and neck cancer patients under 65 years old with low individual SES in disadvantaged neighborhoods conferred a 1.5 to 2-fold higher risk of mortality, compared with patients with high individual SES in advantaged neighborhoods. A cross-level interaction effect was found in lung cancer and breast cancer. Lung cancer and breast cancer patients less than 65 years old with low SES in advantaged neighborhoods carried the highest risk of mortality. Prostate cancer patients aged 65 and above with low SES in disadvantaged neighborhoods incurred the highest risk of mortality. There was no association between SES and mortality for cervical cancer and pancreatic cancer.

**Conclusions:**

Our findings indicate that cancer patients with low individual SES have the highest risk of mortality even under a universal health-care system. Public health strategies and welfare policies must continue to focus on this vulnerable group.

## Introduction

Cancer is a leading cause of death worldwide and it accounted for 7.6 million deaths (13% of all deaths) in 2008 [1]. In Western countries as well as Taiwan, lung cancer, breast cancer, colorectal cancer, prostate cancer, head and neck cancer, cervical cancer and pancreatic cancer are the most common cancers [1], [2], [3]. Cancer treatment is now a serious socioeconomic problem and an important public health issue which deserves more attention.

A growing body of literature suggests a persistent relationship between socioeconomic status (SES) and health status, with SES influencing survival in several common cancers including breast, prostate, and lung cancer and melanoma [4], [5], [6], [7], [8]. Besides individual SES, neighborhood SES influenced the mortality or outcomes [9], [10]. Several studies have explored the combined or cross-level interaction effect of individual SES and neighborhood SES, but the data was conflicting [11], [12], [13]. Different effect of the cross-level interaction of individual SES and neighborhood SES was also observed [14]. Furthermore, no large-scale study has explored the combined effect of individual SES and neighborhood SES on major cancers in the world. Due to limited information on patient survival with different individual SES and neighborhood SES, questions and public health strategies concerning the combined effect of individual and neighborhood SES on cancer survival rates have remained unanswered.

**Table 1:** Baseline characteristics (n = 20488).

Variables	Age <65 years (n = 12382)							Age ≧65 years (n = 8106)						
	High SES		Moderate SES		Low SES		p value	High SES		Moderate SES		Low SES		p value
	(n = 1626)		(n = 2823)		(n = 7933)			(n = 314)		(n = 607)		(n = 7185)		
Mean age, years (±SD)	52	±7.9	51	±8.7	50	±9.2	<0.001	70	±4.8	70	±3.7	75	±6	<0.001
Gender							<0.001							<0.001
Male (%)	965	(59.3)	1322	(46.8)	3618	(45.6)		36	(11.5)	111	(18.3)	2285	(31.8)	
Female (%)	661	(40.7)	1501	(53.2)	4315	(54.6)		278	(88.5)	496	(81.7)	4900	(68.2)	
Urbanization							<0.001							<0.001
Urban (%)	643	(39.5)	1101	(39.0)	2111	(26.6)		133	(42.4)	236	(38.9)	1282	(17.8)	
Suburban (%)	746	(45.9)	1341	(47.5)	3615	(45.6)		129	(41.1)	276	(45.5)	2665	(37.1)	
Rural (%)	237	(14.6)	380	(13.5)	2207	(27.8)		52	(16.6)	95	(15.7)	3238	(45.1)	
Geographic Region							<0.001							<0.001
Northern (%)	966	(16.4)	1628	(57.7)	3288	(41.4)		187	(59.6)	384	(63.3)	2571	(35.8)	
Central (%)	383	(11.8)	727	(25.6)	2138	(26.9)		73	(23.2)	127	(20.9)	2101	(29.2)	
Southern/Eastern (%)	277	(8.5)	471	(16.7)	2511	(31.7)		54	(17.2)	96	(15.8)	2513	(35.0)	
Charlson Comorbidity Index Score							0.564							0.183
0 (%)	909	(55.9)	1627	(57.6)	4533	(57.1)		155	(49.4)	290	(47.8)	3306	(46.0)	
1–6 (%)	519	(31.9)	864	(30.6)	2464	(31.1)		110	(35.0)	218	(35.9)	2657	(37.0)	
>6 (%)	198	(12.2)	332	(11.8)	936	(11.8)		49	(15.6)	99	(16.3)	1222	(17.0)	
Tumor stage							0.026							0.428
Local, locoregional tumor (%)	1418	(87.2)	2529	(89.6)	7100	(89.5)		265	(84.4)	503	(82.9)	6089	(84.7)	
Distant metastatic tumor (%)	208	(12.8)	294	(10.4)	833	(10.5)		49	(15.6)	104	(17.1)	1096	(15.3)	
Surgery							0.017							0.002
Yes (%)	1067	(65.6)	1863	(66.0)	5031	(63.4)		164	(52.2)	305	(50.2)	3262	(45.4)	
No (%)	559	(34.4)	960	(34.0)	2902	(36.6)		150	(47.8)	302	(49.8)	3923	(54.6)	
Nonsurgical Therapy							0.045							0.239
Nil (%)	721	(44.3)	1250	(44.3)	3384	(42.7)		178	(56.7)	350	(57.7)	4570	(63.6)	
Radiotherapy (%)	222	(13.7)	355	(12.6)	1086	(13.7)		48	(15.3)	83	(13.7)	811	(11.3)	
Chemotherapy (%)	354	(21.8)	625	(22.1)	1701	(21.4)		62	(19.7)	102	(16.8)	1160	(16.1)	
Chemoradiotherapy (%)	329	(20.2)	593	(21.0)	1762	(22.2)		26	(8.3)	72	(11.9)	644	(9.0)	
Hospital characteristics														
Teaching level							<0.001							<0.001
Medical center (%)	1133	(69.7)	1922	(68.1)	5262	(66.3)		226	(72.0)	378	(62.3)	4104	(57.1)	
Regional (%)	440	(27.1)	763	(27.0)	2251	(28.4)		77	(24.5)	187	(30.8)	2352	(32.7)	
District (%)	53	(3.3)	138	(4.9)	420	(5.3)		11	(3.5)	42	(6.9)	729	(10.1)	
Ownership							<0.001							<0.001
Public (%)	642	(39.5)	992	(35.1)	2441	(30.8)		149	(47.5)	255	(42.0)	2611	(36.3)	
Non-for-profit (%)	738	(45.4)	1340	(47.5)	3947	(49.8)		119	(37.9)	259	(42.7)	3188	(44.4)	
For-profit (%)	246	(15.1)	491	(17.4)	1545	(19.5)		46	(14.6)	93	(15.3)	1386	(19.3)	

Abbreviation: SES, socioeconomic status.

This article describes and compares the overall survival rates and relative risk of death in patients diagnosed with their first malignant tumor in 2002. The types of cancer included in the study are lung cancer, breast cancer, colorectal cancer, prostate cancer, head and neck cancer, cervical cancer, and pancreatic cancer. We used the Taiwan National Health Insurance Research Database (NHIRD), census data, and public information from the Department of Health to extract individual SES and neighborhood SES data for patients. We used a population-based data set merged with neighborhood SES information to measure the contextual effect of individual and neighborhood SES on major cancer survival rates.

## Materials and Methods

### Ethics Statement

This study was initiated after approval by the Institutional Review Board of Buddhist Dalin Tzu Chi General Hospital, Taiwan. Since all identifying personal information was removed from the secondary files prior to analysis, the review board waived the requirement for written informed consent from the patients involved.

### Database

The data for this study are from the 2002–2007 NHIRD in Taiwan. The NHIRD, which is organized and managed by the National Health Research Institute, is derived from the National Health Insurance Program data. The National Health Insurance Program has been in place in Taiwan since 1995, and it enrolls up to 99% of the Taiwanese population and contracts with 97% of Taiwanese medical providers [15]. The Bureau of National Health Insurance in Taiwan randomly reviews the charts of one per 100 ambulatory and 20 inpatient claim cases and interviews patients in order to verify diagnosis accuracy [16], [17].

The study cohort consisted of patients with incidental lung cancer, breast cancer, colorectal cancer, prostate cancer, head and neck cancer, and cervical cancer who began treatment in 2002.

**Figure 1:**
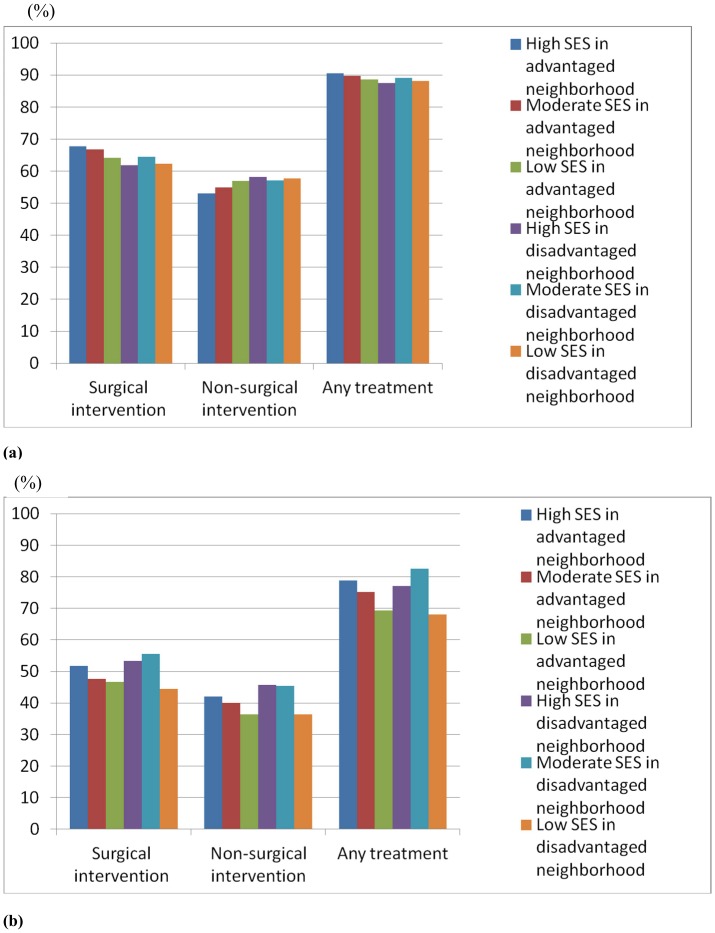
Rates of use of treatment in cancer patient aged less than 65 years (a) and those aged 65 years and above (b).

### Measurement

The key dependent variable of interest was the 5-year survival rate. The overall survival rate was used, because it was not possible to determine cause-specific survival rates based on this registry data. Moreover, Roohan et al. has shown that there is no significant difference between survival models for all-cause-mortality and cancer-specific mortality [18].

The key independent variables were the contextual effects of individual SES and neighborhood SES. Patients were then linked to the mortality data covering the years from 2002 to 2007 to calculate death-free survival time. Each patient was tracked from his or her first curative treatment for a five-year period using administrative data to identify all patients who died during the study period. Patient characteristics included age, gender, geographic location, treatment modality, severity of disease, and monthly income. The disease severity of each patient was based on the modified Charlson Comorbidity Index Score (CCIS), which has been widely accepted in recent years for risk adjustment in administrative claims data sets [19].

### Individual-level Measures

This study used the income-related insurance payment amount as a proxy measure of individual SES at the time of diagnosis, which is an important prognostic factor for cancer [20], [21]. The cancer patients were classified into three groups: (1) low SES: lower than US$571 per month (New Taiwan Dollar (NT$) 20000); (2) moderate SES: between US$571–1141 per month (NT$20000–40000); and (3) high SES: US$1142 per month (NT$40001) or more [22].

### Neighborhood-level Socioeconomic Status

For neighborhood SES, household income is a contextual characteristic representing averages and percentages measured at the enumeration level in the 2001 Taiwan Census.

Neighborhood household income was measured using per capita personal income by township acquired from the 2001 income tax statistics released Taiwan’s Ministry of Finance, (http://www.fdc.gov.tw/dp.asp?mp=5). Advantaged and disadvantaged neighborhoods were distinguished based on the median values for neighborhood characteristics, with advantaged neighborhoods having higher-than-median neighborhood household incomes, and disadvantaged neighborhoods having lower-than-median household incomes.

**Table 2:** Combined effect of individual SES and neighborhood SES on 5-year overall survival rates(n = 20488).

	Neighborhoodsocioeconomic status	Individual socioeconomic status							
		Age <65 years (n = 12382)				Age ≧65 yeasr (n = 8106)			
		High SES	Moderate SES	Low SES		High SES	Moderate SES	Low SES	
		(%)	(%)	(%)	p value	(%)	(%)	(%)	p value
Lung cancer (n = 4698)		n = 1984			0.001	n = 2714			<0.001
	Advantaged	29.4	25.4	20.9		23.6	18.1	20.2	
	Disadvantaged	35.3	25.8	22.5		21.9	28.3	17.0	
Colorectal cancer (n = 5135)		n = 2536			0.005	n = 2599			0.023
	Advantaged	66.7	66.7	59.4		54.1	64.1	53.9	
	Disadvantaged	66.7	67.8	59.0		56.0	44.4	50.3	
Breast cancer (n = 3581)		n = 3223			0.079*	n = 358			0.895
	Advantaged	88.1	85.9	82.9		70.0	85.7	72.4	
	Disadvantaged	87.4	84.3	81.9		66.7	76.9	71.0	
Cervical cancer (n = 1346)		n = 1066			0.262	n = 280			0.263
	Advantaged	85.7	73.8	73.4		50.0	90.0	60.8	
	Disadvantaged	86.7	79.5	78.8		0.0	50.0	51.1	
Prostate cancer (n = 1247)		n = 204			0.143	n = 1043			<0.001
	Advantaged	85.4	77.4	80.0		84.4	73.4	64.5	
	Disadvantaged	84.4	76.5	64.2		80.8	89.3	53.3	
Head and neck cancer (n = 3770)		n = 3053			0.001	n = 717			0.152
	Advantaged	71.5	61.5	55.7		60.0	48.9	50.0	
	Disadvantaged	60.6	59.8	55.3		64.5	44.0	42.1	
Pancreas cancer (n = 711)		n = 316			0.008	n = 395			0.448
	Advantaged	23.7	24.5	36.5		50.0	28.6	16.6	
	Disadvantaged	15.0	31.0	17.9		0.0	20.0	47.8	

Abbreviation: 95% CI, 95% confidence interval.

* In female breast cancer, the p-value of survival rates between the patients in high individual SES in advantaged neighborhoods and patients in low SES in disadvantaged neighborhoods was 0.014.

### Other Variables

The urbanization level of residences were classified in 7 levels based on 5 indices in Taiwan: population density, percentage of residents with college level or higher education, percentage of residents >65 years old, percentage of residents who were agriculture workers, and the number of physicians per 100000 people [23]. We recorded the urbanization level of residences as urban (urbanization level 1), sub-urban (urbanization levels 2–3), or rural (urbanization levels 4–7).

The hospitals were categorized by ownership (public, nonprofit, or for-profit), and hospital level (medical center, regional or district hospital). The geographic regions where the cancer patients resided were recorded as Northern, Central, Southern and Eastern Taiwan.

### Statistical Analysis

The SAS statistical package (version 9.2; SAS Institute, Inc., Cary, NC, USA), and SPSS (version 15, SPSS Inc., Chicago, IL, USA) were used for data analysis. Pearson’s chi-square test was used for categorical variables such as gender, level of urbanization, geographic regions of residence, category of Charlson Comorbidity Index Score, treatment modality, tumor extent, and hospital characteristics (teaching level, ownership, and caseload) in major cancer patients. Continuous variables were analyzed with a one-way ANOVA test. The percentage of patients who underwent surgical intervention/nonsurgical intervention was calculated.

The cumulative 5-year survival rates and the survival curves were constructed and compared using the log-rank test. Survival curves, stratified by individual SES and neighborhood SES, were measured from the time of diagnosis by using overall mortality as the event variable. The Cox proportional hazards regression model adjusting for patients’ characteristics (age, gender, Charlson Comorbidity Index Score, urbanization and area of residence), tumor extent, treatment modality (operation, adjuvant therapy) and hospital characteristics (ownership, teaching level, and caseload) was used to compare outcomes between different SES categories. SES variables were introduced into the Cox model, with the high individual SES and advantaged neighborhood group as the reference group. A two-sided *p*-value (*p*<0.05) was used to determine statistical significance.

**Figure 2:**
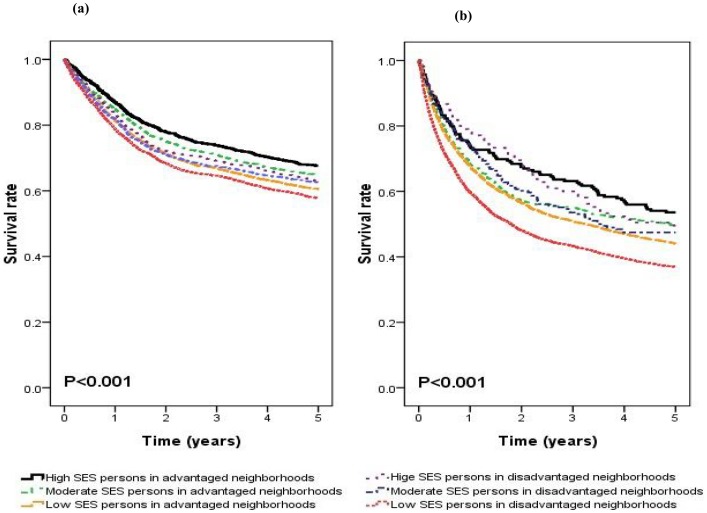
The combined effect of individual and neighborhood SES on cancer survival rates in patients aged less than 65 years (a) and those aged 65 years and above (b).

## Results

### Demographic Data and Clinical Characteristics

A total of 20,488 cancer patients who received treatment were included in the study (Table 1). The mean age at diagnosis differed significantly by individual SES; it was 55 years old for the high individual SES group, 54 years old for the moderate individual SES group, and 62 years old for the low individual SES group. Interaction effects between age and several other variables were noted, and the patients were further stratified into two groups: less than 65 years old, and 65 years old and older.

Patients younger than 65 years old with low individual SES were more likely to reside in rural areas, specifically in southern and eastern Taiwan, and to undergo treatment in regional and district, for-profit and nonprofit hospitals, compared with cancer patients of high individual SES. Patients with low SES were less likely to undergo surgery.

Patients aged 65 and above with low SES were more likely to reside in rural areas, specifically in central, southern, and eastern Taiwan and to undergo treatment at for-profit and nonprofit regional and district hospital. Patients with low SES were less likely to receive surgery.

We examined the use of surgical intervention/non-surgical intervention according to the individual SES and neighborhood SES. Figure 1a (patients aged below 65 years) and 1b (patients aged 65 years and above) shows the percentage of treatment modality. For cancer patients aged below 65, patients in advantaged neighborhood are more likely to underwent surgical intervention (p = 0.002). For cancer patients aged 65 and above, patients with high individual SES were more likely to receive surgery and non-surgical intervention (p<0.001 and 0.004, respectively).

**Table 3:** Hazard ratios of individual SES for mortality in advantaged and disadvantaged neighborhoods*.

	Neighborhoodsocioeconomicstatus	Individual socioeconomic status											
		Age <65 years (n = 12382)						Age ≧65 years (n = 8106)					
		High SES		Moderate SES		Low SES		High SES		Moderate SES		Low SES	
		AdjustedHR	95%CI	AdjustedHR	95% of CI	AdjustedHR	95%CI	AdjustedHR	95%CI	AdjustedHR	95%CI	AdjustedHR	95%CI
Lung cancer (n = 4698)		n = 1984						n = 2714					
	Advantaged	1.00		1.13	(0.90–1.42)	1.36	(1.10–1.67)	1.00		1.03	(0.72–1.48)	0.92	(0.67–1.26)
	Disadvantaged	0.91	(0.68–1.22)	1.24	(0.96–1.60)	1.23	(1.00–1.52)	0.83	(0.51–1.37)	0.84	(0.55–1.29)	1.03	(0.76–1.41)
Colorectal cancer (n = 5135)		n = 2536						n = 2599					
	Advantaged	1.00		1.06	(0.81–1.37)	1.40	(1.11–1.77)	1.00		0.80	(0.51–1.26)	0.96	(0.68–1.37)
	Disadvantaged	0.84	(0.59–1.18)	1.11	(0.81–1.52)	1.45	(1.14–1.83)	1.16	(0.58–2.29)	1.41	(0.88–2.27)	1.07	(0.75–1.53)
Breast cancer (n = 3581)		n = 3223						n = 358					
	Advantaged	1.00		1.38	(0.93–2.05)	1.70	(1.18–2.44)	1.00		0.53	(0.08–3.60)	0.97	(0.26–3.65)
	Disadvantaged	1.06	(0.57–1.94)	1.59	(0.99–2.55)	1.63	(1.13–2.36)	1.71	(0.16–18.15)	1.13	(0.21–6.22)	1.31	(0.34–5.14)
Cervical cancer (n = 1346)		n = 1066						n = 280					
	Advantaged	1.00		1.22	(0.51–2.94)	1.11	(0.48–2.56)	1.00		0.34	(0.03–4.41)	1.42	(0.28–7.18)
	Disadvantaged	0.57	(0.11–2.86)	0.79	(0.31–2.05)	0.87	(0.38–2.01)	11.41	(0.83–156.48)	5.74	(0.46–71.64)	1.27	(0.26–6.17)
Prostate cancer (n = 1247)		n = 204						n = 1043					
	Advantaged	1.00		1.16	(0.37–3.65)	0.80	(0.23–2.78)	1.00		1.99	(0.82–4.81)	1.78	(0.83–3.83)
	Disadvantaged	0.81	(0.23–2.81)	1.64	(0.45–6.01)	1.80	(0.65–4.95)	1.23	(0.39–3.91)	0.79	(0.21–3.08)	2.54	(1.19–5.44)
Head and neck cancer (n = 3770)		n = 3053						n = 717					
	Advantaged	1.00		1.61	(1.16–2.24)	1.98	(1.47–2.67)	1.00		1.55	(0.63–3.85)	1.18	(0.51–2.74)
	Disadvantaged	1.53	(1.04–2.25)	1.84	(1.30–2.59)	2.08	(1.55–2.79)	0.99	(0.30–3.31)	142	(0.54–3.74)	1.55	(0.68–3.58)
Pancreas cancer (n = 711)		n = 316						n = 395					
	Advantaged	1.00		0.89	(0.54–1.47)	0.66	(0.42–1.06)	1.00		1.95	(0.55–6.98)	2.19	(0.68–7.10)
	Disadvantaged	1.15	(0.63–2.10)	0.82	(0.46–1.48)	1.31	(0.84–2.04)	3.94	(0.89–17.46)	2.66	(0.57–12.32)	2.26	(0.71–7.27)

Abbreviation: Adjusted HR, adjusted hazard ratio; 95% CI, 95% confidence interval; SES, socioeconomic status.

* Adjusted for the patients’ diagnosed age, gender, Charlson Comorbidity Index Score, tumor stage (local and locoregional versus distant metastasis), treatment modality (surgery versus without surgery), nonsurgical treatment (radiotherapy, chemotherapy, or chemoradiotherapy), and hospital characteristics (teaching level, and ownership).

**Table 4:** Sociodemographic characteristics by individual and neighborhood socioeconomic status.

	High individual SES		Moderate individual SES		Low individual SES		p value
	Advantaged neighborhood	Disadvantaged neighborhood	Advantaged neighborhood	Disadvantaged neighborhood	Advantaged neighborhood	Disadvantaged neighborhood	
**Age <65 years**							
Number of patients	1032	594	1854	969	3998	3935	
Number of deaths (%)	334(32)	220(37)	649(35)	363(38)	1576(39)	1660(42)	<0.001*
Mean age, mean±SD	51±8	52±8	51±9	51±8	49±9	51±9	<0.001
Male gender, %	563(55)	402(68)	818(44)	504(52)	1627(41)	1991(51)	<0.001*
Education≧high school, %	64%	54%	63%	54%	62%	51%	<0.001*
Median household income, NT$1000	630±77	494±30	610±70	491±32	596±62	480±35	<0.001
Health care-related resources							
Physicians per 10,000 persons, mean±SD	28±23	12±13	25±22	12±12	23±19	11±11	<0.001
Pharmacies, mean±SD	55±35	21±24	50±33	24±25	53±33	19±24	<0.001
**Age ≥ 65 years**							
Number of patients	209	105	411	196	2863	4322	
Number of deaths (%)	97(46)	53(51)	209(51)	103(53)	1600(56)	2725(63)	<0.001*
Mean age, mean±SD	71±5	69±4	70±4	70±3	75±6	75±6	<0.001
Male gender, %	183(88)	95(91)	335(82)	161(82)	2105(74)	2795(65)	<0.001*
Education≧high school, %	64%	53%	64%	54%	62%	49%	<0.001*
Median household income, NT$1000	620±72	489±32	620±76	491±30	598±64	472±36	<0.001
Health care-related resources							
Physicians per 10,000 persons, mean±SD	29±22	12±12	26±22	12±11	21±18	9±10	<0.001
Pharmacies, mean±SD	52±34	18±24	49±33	22±24	45±33	11±18	<0.001

* Pearson’s chi-square test.

### Univariate Survival Analysis

The Figure 2a and 2b demonstrated the 5-year overall survival rate for all major cancer patients. Table 2 shows the 5-year overall survival rate stratified by age and tumor category. Analysis of the combined effect of individual SES and neighborhood SES revealed that, in patients aged below 65 years, mortality rates were worst among those with low individual and low neighborhood SES with colorectal cancer, head and neck cancer, and pancreatic cancer (Appendix S1). There was borderline difference among breast cancer patients. For lung cancer, patients with low SES in advantaged neighborhood had the poorest prognosis. Among patients aged 65 years and above, no statistically significant difference was found in survival rates between the groups except for with lung cancer and prostate cancer (Appendix S2).

### Multivariable Survival Analysis

Interaction effects were noted between patient age and survival rates by SES. The combined effect of individual SES and neighborhood SES remained statistically significant in the Cox proportional hazards regression model, adjusting for other factors in patients under 65 years old. Adjusted hazard ratios revealed that, among patients under 65 years old, lung cancer, and breast cancer in patients with low individual SES in advantaged neighborhoods conferred a 1.4-fold to 1.7 higher risk of death, compared with patients with high individual SES in advantaged neighborhoods (Table 3). Colorectal cancer patients and head and neck cancer patients with low individual SES in disadvantaged neighborhoods incurred a large increase in the risk of mortality (HR = 1.5 and 2.1, respectively). Among older patients, patients with prostate cancer with low individual SES in disadvantaged neighborhoods incurred an increased risk of death (HR = 2.5; 95% CI, 1.2–5.4). No statistically significant differences in mortality rates were found based on SES among older patients with other cancers. There was no significant difference between SES and survival in cervical cancer patients and pancreatic cancer patients. In order to explore whether cancer patients underwent surgical intervention/nonsurgical intervention will influence the outcomes, we add the variable, (surgical intervention or non-surgical intervention) versus no treatment, and Appendix S3 showed that the impact of individual SES and neighborhood SES on cancer mortality was similar to the results of Table 3.

At baselines, cancer patients with low SES in disadvantaged neighborhood had lower resources for health care (e.g. physicians per 10,000 residents and pharmacies) in both age less than 65 years and 65 years and above groups (Table 4). Cancer patients with high SES in advantaged neighborhood had higher level of education (percentage of education≧high school), higher median household income. This analysis supported the idea why we used 9 individual and neighborhood SES groups.

## Discussion

The combined effect of individual and neighborhood SES differed according to tumor site and patient age. Among patients under 65 years old, colorectal cancer, and head and neck patients with low individual SES in disadvantaged neighborhoods incurred a moderate increase in the risk of mortality. A cross-level interaction effect was noted in lung cancer and breast cancer patients. Among lung cancer and breast cancer patients under 65 years old, patients with low SES in advantaged neighborhoods carried the highest risk of mortality. Prostate cancer patients with low individual SES in advantaged neighborhoods incurred a higher risk of mortality in the older age groups. There was no statistically significant difference in mortality rates for cervical cancer and pancreatic cancer.

In Taiwan, the research regarding the association of SES and mortality in general population is limited. In a cross-sectional and longitudinal study, Chiao et al conducted a longitudinal study and revealed the negative effect of perceived economic strain on well-being (life satisfaction, and self-rated health) among older adults aged 60 and above [24]. Pu et al. showed that subjective poor financial satisfaction was found to be related to poorer health and increased mortality in the elderly [25]. Because only older patients were included, results require validation in younger cohort. Previous studies revealed a negative association between SES and cancer survival rates and the impact varied based on tumor site, insurance style, and cancer screening program [26]. In lung cancer, colorectal cancer, breast cancer and head and neck cancer, there was a negative association between SES and cancer survival rates in patients under 65 years old. Several plausible mechanisms may explain this phenomenon. Different neighborhood-level variables seemed to represent different contextual effects, and have different effects on mortality. This may be attributed to inequalities in the utilization of medical resources. Cancer patients with low SES tend to seek medical advice or undergo treatment in regional or district hospitals, for-profit hospitals, and low-caseload hospitals, which were negative prognostic factors in survival rates, and this may be due to unequal hospital resources. Public hospitals and medical centers that can offer surgery, reconstruction, radiotherapy and chemotherapy are primarily located in urban areas and advantaged neighborhoods. However, most of the patients with low SES live in rural areas and are farther away from medical centers. This inequality in the provision of health services may explain the pronounced effects of individual and neighborhood SES on access to specialized cancer services, and the use of evidence-based therapies after the diagnosis of a malignancy. Our results revealed the negative combined effect of individual and neighborhood SES on cancer survival among cancer patients under 65 years old.

Social isolation, depression, and occupational stress are more prevalent among patients with low SES [27], [28], and these factors may further increase the risk of death among patients with low SES. Patients with high SES living in advantaged neighborhoods may have more opportunities to improve their prognosis; they may be able to use their knowledge, money, power, social connections, and other available resources to improve their health status. In contrast, the greater isolation of individuals with low SES may make it more difficult for them to obtain useful opinions or advice from relatives, friends, or acquaintances [28].

SES may interact with biological characteristics of cancer. For example, prostate cancer in lower SES patients demonstrated more aggressive behavior [29]. Skin melanoma patients without signs of chronic sun-induced damage are prone to have mutations in the *BRAF* or *NRAS* gene [30] Breast cancer and colon cancer have been found to be more aggressive in patients with low SES and in minority groups [31], [32]. In Western countries, HPV-associated cancers, such as oral and oropharyngeal cancer, are associated with higher SES [33]. In HPV-related head and neck cancer, E6–7 protein–induced oncogenesis by blocking the p53 protein is associated with better prognosis and has been shown to respond well to radiotherapy and chemotherapy [34].

Physician discrimination may also affect the outcomes of patients with low SES. In treating chest pain, physicians may be less likely to refer black women for catheterization than white men [35]. Furthermore, regarding the discussion of cancer screening results, such as fecal occult blood tests, and prostate specific antigen results, Asian patients were much less likely to have a discussion about the results with the health care provider, compared with the white patients [36]. In rural areas, physicians were less likely to refer hereditary breast cancer patients for advanced genetics services [37].

In Taiwan, there was no negative association between SES and cervical cancer survival rates. The national Pap smear screening program for cervical cancer was launched in 1995, and the screening rate is up to 54% and the incidence of invasive cervical cancer dropped to 16% in 2002. Early diagnosis and early treatment may explain why there was no significant difference in survival rates among the different SES groups.

Pancreatic cancer is a highly lethal cancer. Surgical resection with or without adjuvant therapy provide the potentially curative therapy [38]. However, up to 37% postoperative morbidity and poor survival rates were noted in pancreatic cancer [39]. Recently, gemcitabine with radiotherapy improved survival rates in locally advanced pancreatic cancer [40]. In our series, the overall survival rate for pancreatic cancer was poor. In multivariable analysis, the impact of SES didn’t reach statistical significance. The major influence of survival on pancreatic cancer may be contributed by surgeon experience, tumor characteristics, chemotherapy, target therapy and other factors [40], [41], [42].

Cross-level interaction was noted in lung cancer and breast cancer. Patients with low individual SES in advantaged neighborhoods incurred the highest risk of mortality after adjusting for other risk factors, confounding factors and treatment modality. Several mechanisms may explain this phenomenon. Lower effective income for those patients might explain this observation [43], and social resources which are primarily located in disadvantaged neighborhoods may further hinder those patients from accessing medical services [44].

With the exception of prostate cancer, no statistically significant relationships were found between SES and survival rates in lung cancer, breast cancer, colorectal cancer and head and neck cancer patients 65 years old and older. Previous research has demonstrated similar results in head and neck cancer [21]. Possible mechanisms that explain this include increased competing mortality in older cancer patients and differences in family member support. Cancer patients may have died of the index cancer, or of other conditions such as pneumonia, sepsis, or cardiovascular events. Compared with younger cancer patients, older patients tend to have higher CCI scores, an independent predictor of competing mortality [45]. Additionally, for older patients, the support of family members and interaction with community service organizations may influence outcomes; however, the extent of this influence requires further research [46].

In our series, we identified that colorectal cancer, and head and neck cancer patients under 65 years old with low individual SES in disadvantaged neighborhoods conferred a 1.5 to 2-fold higher risk of mortality. Lung cancer and breast cancer patients less than 65 years old with low SES in advantaged neighborhoods carried the highest risk of mortality. Prostate cancer patients aged 65 and above with low SES in disadvantaged neighborhoods incurred the highest risk of mortality. The insurance payer and provider, such as the Bureau of National Health Insurance in Taiwan, may actively provide more information of cancer treatment, cancer therapy quality and accreditation of each health care institution for the above mentioned cancer patients and cooperate with the social welfare workers to help them once they get the application of incident cancer registry from the medical institutions.

This study has several limitations. First, the diagnosis of cancer, and any comorbidities, was completely dependent on ICD codes. Nonetheless, the National Health Insurance Bureau of Taiwan randomly reviews the charts and interviews patients in order to verify diagnosis accuracy [16], [17]. Second, the database does not contain information on tobacco use or dietary habits, which may be risk factors and prognostic factors for head and neck cancer. Third, cancer stages were not included in the dataset. However, previous studies have revealed no statistically significant associations between SES and tumor stage at diagnosis in oral cancer, colorectal cancer, breast cancer, and lung cancer [21], [47], [48], [49], [50].

Our findings indicate the importance of the combined effect of individual SES and neighborhood SES. Public health strategies should focus on patients with low individual SES in order to reduce health disparities.

## Supporting Information

Appendix S1 The combined effect of individual and neighborhood SES on cancer survival rates in patients aged less than 65 years with stratification by tumor.
(DOC)

Appendix S2 The combined effect of individual and neighborhood SES on cancer survival rates in patients aged 65 years and above with stratification by tumor.
(DOC)

Appendix S3 Adjusted hazard ratios of individual SES for mortality in advantaged and disadvantaged neighborhoods.
(DOC)
