# Supplementary material for: The Combined Effect of Individual and Neighborhood Socioeconomic Status on Cancer Survival Rates
Source: PLoS One. 2012 Aug 30;7(8):e44325. doi: 10.1371/journal.pone.0044325 (PMC3431308; doi:10.1371/journal.pone.0044325)
Supplement: Appendix S1 — The combined effect of individual and neighborhood SES on cancer survival rates in patients aged less than 65 years with stratification by tumor. (DOC) [file pone.0044325.s001.doc]

1. **Lung Cancer (n=1984)**

p=0.001

1. **Colorectal cancer (n=2536)**

p=0.005

**(c) Breast cancer (n=3223)**

p=0.079

1. **Cervical cancer (n=1066)**

p=0.262

1. **Prostate cancer (n=204)**

p=0.143

1. **Head and neck cancer (n=3053)**

p=0.001

1. **Pancreas cancer (n=316)**

p=0.008

**Appendix S1.**

The combined effect of individual and neighborhood SES on cancer survival rates in patients aged less than 65 years.
