# Supplementary material for: The Combined Effect of Individual and Neighborhood Socioeconomic Status on Cancer Survival Rates
Source: PLoS One. 2012 Aug 30;7(8):e44325. doi: 10.1371/journal.pone.0044325 (PMC3431308; doi:10.1371/journal.pone.0044325)
Supplement: Appendix S2 — The combined effect of individual and neighborhood SES on cancer survival rates in patients aged 65 years and above with stratification by tumor. (DOC) [file pone.0044325.s002.doc]

1. **Lung Cancer (n=2714)**

p<0.001

1. **Colorectal cancer (n=2599)**

p=0.023

1. **Breast cancer ( n=358)**

p=0.895

1. **Cervical cancer (n=280)**

p=0.263

1. **Prostate cancer (n=1043)**

p<0.001

1. **Head and neck cancer (n=717)**

p=0.152

1. **Pancreas cancer (n=395)**

p=0.448

**Appendix S2.**

The combined effect of individual and neighborhood SES on cancer survival rates in patients aged 65 years and above.
