# Supplementary material for: The Combined Effect of Individual and Neighborhood Socioeconomic Status on Cancer Survival Rates
Source: PLoS One. 2012 Aug 30;7(8):e44325. doi: 10.1371/journal.pone.0044325 (PMC3431308; doi:10.1371/journal.pone.0044325)
Supplement: Appendix S3 — Adjusted hazard ratios of individual SES for mortality in advantaged and disadvantaged neighborhoods. (DOC) [file pone.0044325.s003.doc]

| **Appendix S3.** Hazard ratios of individual SES for mortality in advantaged and disadvantaged neighborhoods. | | | | | | | | | | | | | | | | |
| --- | --- | --- | --- | --- | --- | --- | --- | --- | --- | --- | --- | --- | --- | --- | --- | --- |
| Site of cancer | Neighborhood  Socioeconomic  (income) | Individual socioeconomic (Monthly income) | | | | | | | | | | | | | | |
|  |  | Age <65 year old (n=12382) | | | | | | Age ≧65 year old (n=8106) | | | | | | | | |
|  |  | High SES | | Moderate SES | | Low SES | | High SES | | | Moderate SES | | | Low SES | | |
|  |  | Adjusted  HR | 95% CI | Adjusted  HR | 95% CI | Adjusted  HR | 95% CI | Adjusted  HR | | 95% CI | Adjusted  HR | | 95% CI | Adjusted  HR | | 95% CI |
| Lung cancer (n=4698) | | n=1984 | | | | | | n=2714 | | | | | | | | |
|  | Advantaged | 1.00 |  | 1.09 | (0.87-1.37) | 1.37 | (1.11-1.68) | 1.00 |  | | 1.17 | (0.82-1.68) | | 0.97 | (0.71-1.33) | |
|  | Disadvantaged | 0.89 | (0.66-1.17) | 1.18 | (0.91-1.52) | 1.24 | (1.01-1.52) | 0.87 | (0.53-1.43) | | 0.94 | (0.61-1.44) | | 1.10 | (0.81-1.51) | |
| Colorectal cancer (n=5135) | | n=2536 | | | | | | n=2599 | | | | | | | | |
|  | Advantaged | 1.00 |  | 1.10 | (0.85-1.43) | 1.50 | (1.18-1.89) | 1.00 |  | | 0.74 | (0.47-1.16) | | 0.92 | (0.64-1.31) | |
|  | Disadvantaged | 1.00 | (0.71-1.40) | 1.16 | (0.84-1.59) | 1.56 | (1.23-1.98) | 1.13 | (0.57-2.24) | | 1.32 | (0.82-2.12) | | 0.99 | (0.70-1.41) | |
| Breast cancer (n=3581) | | n=3223 | | | | | | n=358 | | | | | | | | |
|  | Advantaged | 1.00 |  | 1.25 | (0.84-1.85) | 1.55 | (1.08-2.22) | 1.00 |  | | 0.47 | (0.07-2.99) | | 0.97 | (0.27-3.39) | |
|  | Disadvantaged | 1.04 | (0.57-1.91) | 1.50 | (0.94-2.40) | 1.54 | (1.06-2.22) | 2.35 | (0.22-24.81) | | 1.03 | (0.19-5.52) | | 1.16 | (0.32-4.16) | |
| Cervical cancer (n=1346) | | n=1066 | | | | | | n=280 | | | | | | | | |
|  | Advantaged | 1.00 |  | 1.80 | (0.75-4.29) | 1.73 | (0.76-3.95) | 1.00 |  | | 0.19 | (0.02-2.33) | | 0.98 | (0.19-4.98) | |
|  | Disadvantaged | 0.84 | (0.17-4.18) | 1.08 | (0.42-2.76) | 1.35 | (0.59-3.10) | 4.67 | (0.35-61.86) | | 1.89 | (0.16-22.17) | | 0.86 | (0.18-4.15) | |
| Prostate cancer (n=1247) | | n=204 | | | | | | n=1043 | | | | | | | | |
|  | Advantaged | 1.00 |  | 1.40 | (0.47-4.20) | 0.80 | (0.24-2.67) | 1.00 |  | | 1.85 | (0.77-4.46) | | 1.89 | (0.88-4.05) | |
|  | Disadvantaged | 0.92 | (0.27-3.14) | 1.62 | (0.44-5.96) | 1.72 | (0.64-4.64) | 1.49 | (0.47-4.70) | | 0.71 | (0.18-2.76) | | 2.35 | (1.10-5.01) | |
| Head and neck cancer (n=3770) | | n=3053 | | | | | | n=717 | | | | | | | | |
|  | Advantaged | 1.00 |  | 1.65 | (1.19-2.29) | 1.97 | (1.46-2.66) | 1.00 |  | | 1.50 | (0.61-3.68) | | 1.23 | (0.54-2.84) | |
|  | Disadvantaged | 1.59 | (1.08-2.33) | 1.77 | (1.25-2.49) | 1.97 | (1.47-2.65) | 0.95 | (0.29-3.13) | | 1.57 | (0.60-4.11) | | 1.60 | (0.71-3.64) | |
| Pancreas cancer (n=711) | | n=316 | | | | | | n=395 | | | | | | | | |
|  | Advantaged | 1.00 |  | 1.02 | (0.62-1.68) | 0.72 | (0.46-1.15) | 1.00 |  | | 2.11 | (0.59-7.50) | | 2.27 | (0.70-7.32) | |
|  | Disadvantaged | 1.14 | (0.62-2.09) | 0.81 | (0.45-1.46) | 1.43 | (0.92-2.22) | 3.52 | (0.80-15.49) | | 3.23 | (0.70-14.83) | | 2.40 | (0.75-7.70) | |
| * Adjusted variables were the patients diagnosed age, gender, CCIS categories, tumor stage (local and locoregional versus distant metastasis), treatment (with treatment versus without treatment), and hospital characteristics (teaching level, ownership). | | | | | | | | | | | | | | | | |
